# Supplementary material for: Does the Digital Economy Promote Dietary Diversity Among Chinese Residents?
Source: Foods. 2025 Nov 13;14(22):3873. doi: 10.3390/foods14223873 (PMC12650907; doi:10.3390/foods14223873)
Supplement: Supplementary file 1 [file foods-14-03873-s001.zip › foods-3941090-supplementary.pdf]

1. Dietary balance index constitution

Table S1. Components of DBI_22 [1,2].

Subgroup	Score	Intake range by energy intake level										
		1000 kcal	1200 kcal	1400 kcal	1600 kcal	1800 kcal	2000 kcal	2200 kcal	2400 kcal	2600 kcal	2800 kcal	3000 kcal
Cereal	-12~12 [#]	0g=-12<br>75~95g=0<br>>170g=12	<15g=-12<br>90~110g=0<br>>185g=12	0g=-12<br>125~175g=0<br>>300g=12	<10g=-12<br>175~225g=0<br>>390g=12	<35g=-12<br>200~250g=0<br>>415g=12	<5g=-12<br>225~275g=0<br>>495g=12	<30g=-12<br>250~300g=0<br>>520g=12	0g=-12<br>275~325g=0<br>>600g=12	<50g=-12<br>325~375g=0<br>>650g=12	<75g=-12<br>350~400g=0<br>>675g=12	<100g=-12<br>375~425g=0<br>>700g=12
Vegetable	-6~0	≥200g=0<br>160~199g=-1<br>Score decreased 1 with intake amount decreased 40g<br>0g=-6	≥250g=0<br>200~249g=-1<br>Score decreased 1 with intake amount decreased 50g<br>0g=-6	≥300g=0<br>240~299g=-1<br>Score decreased 1 with intake amount decreased 60g<br>0g=-6		≥400g=0<br>320~399g=-1<br>Score decreased 1 with intake amount decreased 80g<br>0g=-6	≥450g=0<br>360~449g=-1<br>Score decreased 1 with intake amount decreased 90g<br>0g=-6		≥500g=0<br>400~499g=-1<br>Score decreased 1 with intake amount decreased 100g<br>0g=-6			≥600g=0<br>480~599g=-1<br>Score decreased 1 with intake amount decreased 120g<br>0g=-6
Fruit	-6~0	≥150g=0; 120~149g=-1<br>Score decreased 1 with intake amount decreased 30g<br>0g=-6			≥200g=0; 160~199g=-1<br>Score decreased 1 with intake amount decreased 40g<br>0g=-6		≥300g=0; 240~299g=-1<br>Score decreased 1 with intake amount decreased 60g<br>0g=-6		≥350g=0; 280~349g=-1<br>Score decreased 1 with intake amount decreased 70g<br>0g=-6		≥400g=0; 320~399g=-1<br>Score decreased 1 with intake amount decreased 80g<br>0g=-6	

Dairy	-6~0	≥500g=0<br>Score decreased 1 with intake amount decreased 100g<br>0g=-6		≥350g=0<br>Score decreased 1 with intake amount decreased 70g<br>0g=-6	≥300g=0<br>Score decreased 1 with intake amount decreased 60g<br>0g=-6		
Soybean	-6~0	≥5g=0<br>Score decreased 1 with intake amount decreased 1g<br>0g=-6	≥15g=0<br>Score decreased 1 with intake amount decreased 3g<br>0g=-6			≥25g=0<br>Score decreased 1 with intake amount decreased 5g<br>0g=-6	
Red meat and products, Poultry and game	-4~4	0g=-3<br>1~5g=-2<br>6~10g=-1<br>11~20g=0<br>21~25g=1<br>26~30g=2<br>31~35g=3<br>>35g=4	0g=-4<br>1~5g=-3<br>6~10g=-2<br>11~15g=-1<br>16~35g=0<br>36~40g=1<br>41~45g=2<br>46~50g=3	0g=-4<br>1~10g=-3<br>11~20g=-2<br>21~30g=-1<br>31~50g=0<br>51~60g=1<br>61~70g=2<br>71~80g=3	0g=-4<br>1~15g=-3<br>16~30g=-2<br>31~45g=-1<br>46~55g=0<br>56~70g=1<br>71~85g=2<br>85~100g=3	0g=-4<br>1~20g=-3<br>21~40g=-2<br>41~60g=-1<br>61~90g=0<br>91~110g=1<br>111~130g=2<br>131~150g=3	0g=-4<br>1~25g=-3<br>26~50g=-2<br>51~75g=-1<br>76~125g=0<br>126~150g=1<br>151~175g=2<br>176~200g=3

			>50g=4	>80g=4		>100g=4		>150g=4	>200g=4	
Fish and	-4~0	0g=-4	<5g=-4	<10g=-4		<5g=-4		0g=-4	<25g=-4	<50g=-4
shrimp		1~4g=-3	5~9g=-3	10~19g=-3		5~19g=-3		1~24g=-3	25~49g=-3	50~74g=-3
		5~9g=-2	10~14g=-2	20~29g=-2		20~34g=-2		25~49g=-2	50~74g=-2	75~99g=-2
		10~14g=-1	15~19g=-1	30~39g=-1		35~49g=-1		50~74g=-1	75~99g=-1	100~124g=-
		≥15g=0	≥20g=0	≥40g=0		≥50g=0		≥75g=0	≥100g=0	1
										≥125g=0
Egg	~4-4	0g=-4	<5g=-4		0g=-4		0g=-4			
		1~5g=-3	6~10g=-3		1~10g=-3		1~15g=-3			
		6~10g=-2	11~15g=-2		11~20g=-2		16~30g=-2			
		11~15g=-1	16~20g=-1		21~30g=-1		31~45g=-1			
		16~25g=0	21~30g=0		31~50g=0		46~55g=0			
		26~30g=1	31~35g=1		51~60g=1		56~70g=1			
		31~35g=2	36~40g=2		61~70g=2		71~85g=2			
		36~40g=3	41~45g=3		71~80g=3		86~100g=3			
		>40g=4	>45g=4		>80g=4		>100g=4			
Cooking	0~6##	≤20g=0	≤25g=0					≤30g=0	≤35g=0	
oil		21~25g=1	26~30g=1					31~35g=1	36~40g=1	
		>45g=6	>50g=6					>55g=6	>60g=6	
Alcoholic	0~6	Male:≤25g=0, 26-40g=1, 6~100g score increased 1 with intake amount increased 15g, >100g=6								
beverage		(25g alcohol=750ml beer or 250ml wine or 75g liquor (38°) or 50g liquor > 38°)								
		Female: ≤15g=0,16g=1, 16-25g=1, score increased 1 with intake amount increased 10g, >65g=6								
		(15g alcohol=450ml beer or 150ml wine or 50g liquor (38°) or 30g liquor (> 38°))								
Addible	0~6	≤25g=0; 26g=1; Score increased 1 with intake amount increased 5g; >50g=6								
sugar										
Salt	0~6	<2g=0	<3g=0	<4g=0	<5g=0					

		2~3g=1<br>Score increased 1 with intake amount increased 2g<br>>12g=6	3~4g=1<br>Score increased 1 with intake amount increased 2g<br>>13g=6	4~5g=1<br>Score increased 1 with intake amount increased 2g<br>>14g=6	5~6g=1<br>Score increased 1 with intake amount increased 2g<br>>15g=6
Diet variety	-12~0	≥12 kinds of food (soybean is 5g)=0, score decreased 1 with decreased 1 kinds of food, 0 kinds of food = -12			
Drinking water	-12~0	≥1200 ml=0, score decreased 1 with intake amount decreased 100 ml, <100 ml= -12			

*If the energy is between the two levels, the method used for the lower energy level. If the score of the food com ponent corresponding to the energy level is empty, it indicates that the value is taken in the same way as the previous energy level. ** Cereal include rice, wheat, dried legumes (exclude soybean) and tubers. Intake amount means fresh amount. Sweet potato: intake amount divided by 3, potato: intake amount divided by 4, yam and yam bean: divided by 6. # The score increased (decreased) by 2 for each 1 unit weight intake increased (decreased) when the energy intake level is 1000-1400kcal, and by 1 for each 1 unit weight intake increased (decreased) when the energy intake level is over 1600 kcal. Unit weights are 15g when the energy levels are1000-1200 kcal and 1600-1800 kcal, 20g when the energy level is 2000-2200kcal and 25g when the energy levels are 1400kcal and over 2400 kcal. ## score increased 1 with intake amount increased 5g from score 1 to 6. ### 25g alcohol=650ml beer (4% alcohol) or 250ml wine (12% alcohol) or 75ml low degree liquor (38% alcohol) or 50ml high degree liquor (52%alcohol); 15g alcohol=400ml beer or 150ml wine or 50ml low degree liquor or 30ml high degree liquor, 10g alcohol=250ml beer or 100ml wine or25ml low degree liquor or 20ml high degree.

2. Calculation of digital economy index

The computation of the digital economy index is executed as follows: Initially, a principal component analysis (PCA) applicability test is conducted subsequent to the standardization of eight selected digital economy indicators. The Kaiser-Meyer-Olkin (KMO) measure of sampling adequacy yields a value of 0.828, and the p-value from Bartlett's test of sphericity is 0.000, implying that these indicators are appropriate for PCA. Secondly, pertinent principal components are derived from the variance decomposition of the data. Based on the eigenvalues and cumulative variance contribution rate, the first two principal components exhibit values greater than 1 and a cumulative variance contribution rate of 81.329%. This suggests that these components possess stronger explanatory power and can encapsulate the majority of the original data's information; hence, they are designated as W_1 and W_2, respectively. Subsequently, using the component score coefficient matrix (Table S2) alongside the standardized values of each digital economy indicator enables the calculation of the principal component scores, as articulated in the succeeding equations.

$$C_1 = 0.164Z_1 + 0.180Z_2 + 0.092Z_3 + 0.179Z_4 + 0.150Z_5 + 0.148Z_6 + 0.177Z_7 + 0.112Z_8 \quad (S.1.)$$

$$C_2 = 0.230Z_1 - 0.143Z_2 + 0.679Z_3 - 0.201Z_4 - 0.371Z_5 + 0.275Z_6 + 0.019Z_7 - 0.242Z_8 \quad (S.2.)$$

Table S2. Component score coefficient matrix.

Indicators	C_1	C_2
Z-Score: business volume of post and telecommunications	0.164	0.230
Z-Score: number of mobile telephone subscribers at year-end	0.180	-0.143
Z-Score: number of landline telephone subscribers at year-end	0.092	0.679
Z-Score: Internet population	0.179	-0.201
Z-Score: Internet penetration rate	0.150	-0.371
Z-Score: number of employees in the information-related industry	0.148	0.275
Z-Score: Website counts	0.177	0.019
Z-Score: number of .cn domain	0.112	-0.242

The proportion of variance attributable to each principal component served as the weight in a linearly weighted approach to compute the composite score for the two principal components, as delineated in the subsequent equation:

$$Score_i = W_1C_1 + W_2C_2 \quad (S.3.)$$

Ultimately, the computed digital economy index scores exhibited negative values, which were not conducive to the requirements of the study. Consequently, the definitive digital economy index was derived by normalizing the principal component composite score.

$$Index_i = \left[\frac{Score_i}{max(Score_i) - min(Score_i)} \times 0.4 + 0.6\right] \times 100 \quad (S.4.)$$

In the aforementioned equation, $Index_i$ represents the digital economy index, while $Score_i$ denotes the principal component composite score. The term max ($Score_i$) signifies the

maximum value of the composite score, and min($Score_i$) represents its minimum value. The transformed digital economy index is scaled to fall within the interval [0, 100]. The upward trends in China's digital economy across nine provinces throughout the survey period are depicted in Figure S1.

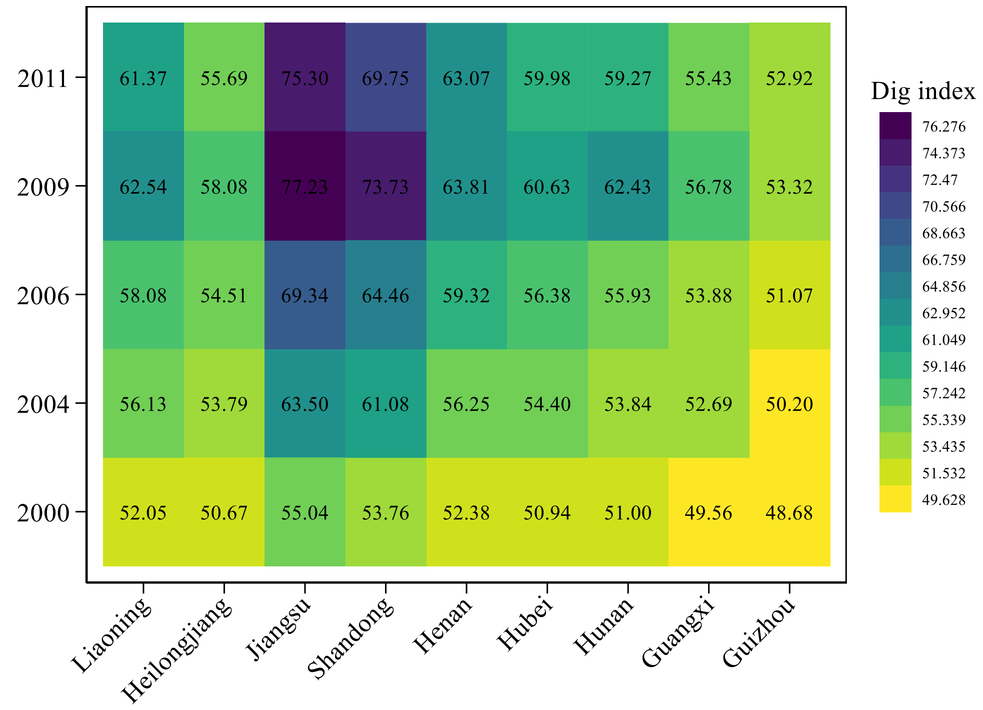

Figure S1. Regional and temporal variation in digital economy index across nine provinces, 2000-2011.

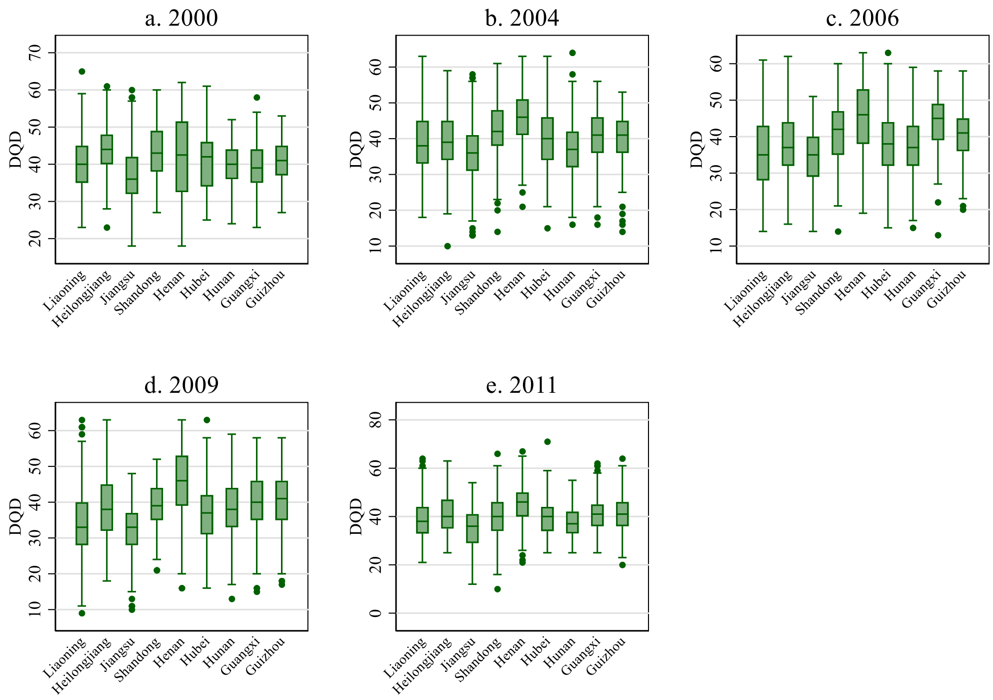

Figure S2. Regional and temporal variation in DQD across nine provinces, 2000-2011.

3. Calculation of industry transformation

The Industrial Structure Theil Index, a robust gauge of industrial structure rationalization, is articulated as follows:

$$TL_{i,t} = \sum_{n=1}^{3} y_{i,n,t} \times ln\left(y_{i,n,t}/l_{i,n,t}\right) \qquad (S.5.)$$

Let $y_{i,n,t}$ represent the proportion of the added value of industry n to the GDP of region i in year t, and let $l_{i,n,t}$ denote the share of employment in industry n as a fraction of the total employment in region i during year t, where n = 1, 2, 3 corresponds to the primary, secondary, and tertiary industries, respectively. The Industrial Structure Theil Index integrates both the value added by industries and employment data to quantify the rationalization of the industrial structure. A Theil Index value of zero indicates a balanced industrial structure. Conversely, a higher index value indicates a more skewed and thus less rationalized structure.

4. Two clustering dietary pattern

Figure S3 presents the average daily intake of 12 food groups for the two dietary patterns and the overall sample. A t-test for mean differences was conducted for each food group, and the results showed statistically significant differences in 11 of the 12 groups, except for alcohol. Cereal, vegetable, and meat were the three food groups with the highest daily intake across the sample. Specifically, cereal intake in Type I was lower than that in both Type II and the full sample, whereas vegetable and meat intake in Type I exceeded those of Type II and the full sample. Regarding high-nutrient-density foods, the consumption of fruits, fish, egg, dairy

products, and soybean in Type I was significantly higher than in Type II. Similarly, Type I also showed higher intake levels of oil, salt, and sugar. Overall, the dietary characteristics of Type I resemble a modern Western dietary pattern typical of more economically developed regions [3], whereas Type II remains grain-dominant, reflecting characteristics of the traditional Chinese diet.

To further assess the quality of the two dietary patterns, we employed the previously calculated DBI. Table S3 reports the descriptive statistics for four indicators—DQD, HBS, LBS, and DDS—across the two patterns. The t-test results indicated statistically significant differences in all four indicators. The DQD value of Type I was lower than that of Type II, suggesting a more balanced dietary structure. Specifically, Type I exhibited a lower HBS, indicating less excessive food intake, and higher LBS and DDS, reflecting lower levels of insufficient intake and greater dietary diversity. In summary, based on food intake and dietary balance analyses, Type I demonstrates superior dietary diversity and overall quality compared with Type II.

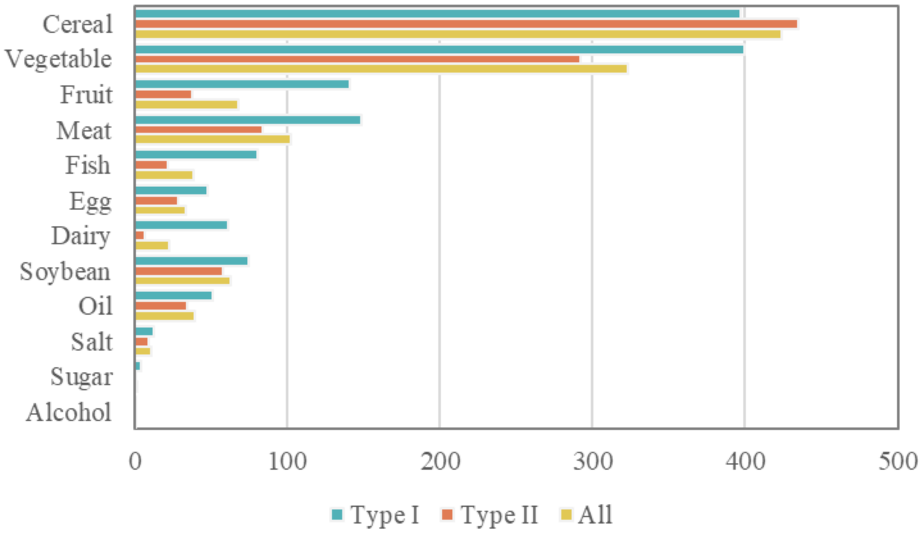

Figure S3. Mean share of dietary food intake under two clustering dietary patterns (g).

Table S3. Descriptive analysis of dietary balance index by dietary patterns.

Type	DQD	HBS	LBS	DDS
I	33.388	14.931	-13.800	-3.715
	(8.207)	(5.473)	(8.794)	(1.478)
II	41.506	15.526	-21.123	-5.443
	(7.710)	(4.930)	(8.902)	(1.561)
P value	0.000***	0.000***	0.000***	0.000***

5. Calculation of entropy and Simpson index

The calculation of entropy is based on the following formula, as referenced by established methodology [4], incorporating the consumption share w_i. The term w_i is defined as the average daily category of food consumed, divided by the total number of food items consumed on three days. An increase in Entropy values signifies enhanced dietary diversity. Theoretically, when logn reaches its maximum, the distribution of food consumption shares becomes equitable across all food varieties.

$$E = \sum_{i=1}^{n} w_i \log\left(\frac{1}{w_i}\right) \quad \text{(S. 6.)}$$

The equation of Simpson index is as follows, and share w_i is defined as above. The Simpson index ranges from 0 to 1- 1/n, where a value of 0 signifies the consumption of only one food item, and the maximum value indicates that all food items are consumed equally.

$$SI = 1 - \sum_{i=1}^{n} w_i^2 \quad (S.7.)$$

References

1. He,Y.N.; Fang, Y.H.; Xia, J. Update of the Chinese diet balance index: DBI_16. *Acta Nutr. Sin.* **2018**, *40*, 526–530.
2. He, Y.N.; Ye, C.; Fang, Y.H.; Lian, Y.Y. Update of the Chinese diet balance index: DBI_22. *Acta Nutr. Sin.* **2024**, *46*, 209–214.
3. Zhu, M.R.; Zong, J. Shifting to a recommended dietary pattern could promote sustainable development of the environment and human health. *Front. Agric. Sci. Eng.* **2023**, *10*, 73–82.
4. Theil, H.; Finke, R. The consumer's demand for diversity. *Eur. Econ. Rev.* **1983**, *23*, 395-400.
